# Moderate Photoinhibition of Photosystem II Significantly Affects Linear Electron Flow in the Shade-Demanding Plant *Panax notoginseng*

**DOI:** 10.3389/fpls.2018.00637

**Published:** 2018-05-15

**Authors:** Wei Huang, Shi-Bao Zhang, Tao Liu

**Affiliations:** ^1^Key Laboratory of Economic Plants and Biotechnology, Kunming Institute of Botany, Chinese Academy of Sciences, Kunming, China; ^2^National Local Joint Engineering Research Center on Germplasm Utilization and Innovation of Chinese Medicinal Materials in Southwest China, Yunnan Agricultural University, Kunming, China

**Keywords:** linear electron flow, lumenal acidification, photoinhibition, photosystem II, proton motive force

## Abstract

Although photoinhibition of photosystem II (PSII) frequently occurs under natural growing conditions, knowledge about the effect of moderate photoinhibition on linear electron flow (LEF) remains controversial. Furthermore, mechanisms underlying the decrease in LEF upon PSII photoinhibition are not well clarified. We examined how selective PSII photoinhibition influenced LEF in the attached leaves of shade-demanding plant *Panax notoginseng*. After leaves were exposed to a high level of light (2258 μmol photons m^-2^ s^-1^) for 30 and 60 min, the maximum quantum yield of PSII (*F*_v_/*F*_m_) decreased by 17 and 23%, respectively, whereas the maximum photo-oxidizable P700 (*P*_m_) remained stable. Therefore, this species displayed selective PSII photodamage under strong illumination. After these treatments, LEF was significantly decreased under all light levels but acidification of the thylakoid lumen changed only slightly. Furthermore, the decrease in LEF under low light was positively correlated with the extent of PSII photoinhibition. Thus, the decline in LEF was not caused by the enhancement of lumenal acidification, but was induced by a decrease in PSII activity. These results indicate that residual PSII activity is an important determinant of LEF in this shade-adapted species, and they provide new insight into how strong illumination affects the growth of shade-demanding plants.

## Introduction

During oxygenic photosynthesis in plants, leaves absorb light energy to drive photosynthetic electron flow in the chloroplasts. In linear electron flow (LEF), electrons from the splitting of water by the oxygen-evolving complex (OEC) are transferred to NADP^+^, reducing NADP^+^ to NADPH. This transfer is coupled to proton translocation across the thylakoid membrane, from stroma to lumen, which generates proton motive force (*pmf*) to power the production of ATP via ATP synthase. Although light drives photosynthesis in higher plants, excessive irradiance can cause photoinhibition in the chloroplasts. Photosystem II (PSII) is very sensitive to high-light stress because D1 protein is decreased in the PSII reaction centers and OEC activity is subjected to photodamage (Aro et al., 1993a,b; Hakala et al., 2005; Ohnishi et al., 2005; Takahashi et al., 2009). This phenomenon, photoinhibition of PSII, can influence photosynthetic electron flow and overall plant growth (Kulheim et al., 2002; Murchie and Niyogi, 2011; Tikkanen et al., 2014).

Although the effect of PSII photoinhibition on LEF has been examined, the conclusions have been inconsistent from those studies. For example, a moderate decline in PSII activity does not result in a decrease in the light-saturated rates of CO_2_ assimilation for leaves from *Salix* sp. (Ogren and Sjostrom, 1990), or cause a reduction in the rate of oxygen evolution by *Capsicum annuum* leaves (Lee et al., 1999). Kornyeyev et al. (2006) have reported that moderate PSII inactivation (<50%) is not associated with decreases in LEF and CO_2_ assimilation at light intensities above 150 μmol photons m^-2^ s^-1^. In contrast, a correlation between PSII photoinhibition and the rate of electron transport has been observed with leaves of *Chenopodium album* in the presence of an inhibitor of PSII repair (Hikosaka et al., 2004), and also with leaves of *Oryza sativa* that are exposed to different periods of combined chilling–light stress (Hirotsu et al., 2005). Furthermore, Tikkanen et al. (2014) have stated that electron transfer from PSII to PSI is depressed upon moderate PSII photoinhibition. Therefore, these contrasting results underscore the need for further research that can clearly elucidate the effect of selective PSII photoinhibition on LEF.

In previous studies that explored how PSII photoinhibition influences LEF, some factors of photosynthesis have been concomitant, such as the closure of stomata in response to low temperatures (Hirotsu et al., 2005), chilling-induced PSI photoinhibition (Kornyeyev et al., 2006), PSI photoinhibition due to intense illumination (Tikkanen et al., 2014), and measurements being made with detached leaves (Hikosaka et al., 2004; Kornyeyev et al., 2006; Tikkanen et al., 2014). Under low-temperature stress, a longer treatment period means that PSII photoinhibition will be more severe and accompanied by a larger decrease in stomatal conductance (Hirotsu et al., 2005), thereby affecting the rate of the Calvin-Benson cycle and, thus, LEF. Kornyeyev et al. (2006) have conducted experiments with still-attached *Arabidopsis thaliana* leaves that are initially exposed for 2 h to 5°C and intense light (2000 μmol photons m^-2^ s^-1^). They have found that this combined chilling–light treatment leads to a 45% decrease in PSI activity. Likewise, Tikkanen et al. (2014) have shown that a high-light treatment causes significant PSI photoinhibition in *pgr5*-plants of *A. thaliana*. Because PSI photoinhibition can severely affect the rate of LEF (Zivcak et al., 2015), the conclusions of Kornyeyev et al. (2006) and Tikkanen et al. (2014) are interfered by PSI photoinhibition. Some research has utilized detached, rather than attached, leaves for making photosynthetic measurements (Hikosaka et al., 2004; Kornyeyev et al., 2006; Tikkanen et al., 2014). This decision has cast doubt on how stomatal conductance might be influenced in those experiments. Therefore, it is difficult to confirm those previous results due to the shortcomings associated with the investigations.

Although PSII photoinhibition diminishes LEF under low light (Kornyeyev et al., 2006; Tikkanen et al., 2014), the responsible mechanism is unclear. Previous reports have indicated that moderate PSII photoinhibition can lead to a significant decrease in LEF (Tikkanen et al., 2014) and over-acidification of the thylakoid lumen is associated with down-regulation of LEF (Livingston et al., 2010). Specifically, it is unknown whether the decrease in LEF upon moderate PSII photoinhibition is caused by a decline in PSII activity or by the enhancement of lumenal acidification. When PSII is photo-damaged, cyclic electron flow (CEF) is significantly stimulated when plants are allowed to recover under low light (Huang et al., 2010, 2016a), which might make the thylakoid lumen more acidic. Lumenal acidification plays a critical role in controlling electron transfer from PSII at the Cyt *b*_6_/*f* (Nishio and Whitmarsh, 1993; Hope et al., 1994; Takizawa et al., 2007). In *hcef1*, an *A. thaliana* mutant with high CEF, increased NDH-dependent CEF leads to over-acidification of the thylakoid lumen, thus restricting LEF (Livingston et al., 2010). Under drought stress without PSII photoinhibition, the stimulation of CEF increases the lumenal acidification, enhancing NPQ and lowering LEF in the resurrection plant *Paraboea rufescens* (Huang et al., 2012). The induction of NPQ under high light does not strongly decrease LEF, as shown by the similar light-intensity dependence of photosynthesis in WT, *npq1*, and *npq4* (Takahashi et al., 2009; Johnson and Ruban, 2010). Therefore, a change in CEF stimulation may affect the operation of LEF. However, it is unclear whether the depression of LEF upon PSII photoinhibition is caused by the loss of PSII activity or the enhancement of lumenal acidification.

In a previous investigation, we observed that shade-grown *Panax notoginseng* exhibited selective PSII photoinhibition after short-term treatment under high light. Therefore, we used plants of this species here to examine the effect of selective PSII photoinhibition on LEF. In this study, we focused on the mechanism underlying the decrease in LEF, and we investigated the relationship between low-light LEF and PSII activity. Regardless of light levels, moderate PSII photoinhibition significantly affected LEF. Furthermore, the reduction in LEF was independent of *pmf* but was significantly related to the decrease in PSII activity.

## Materials and Methods

### Plant Materials

Two-year-old plants of the shade-adapted *P. notoginseng* (Burkill) F. H. Chen ex C. Chow and W. G. Huang were grown indoors under 10% sunlight, and were watered and fertilized normally to avoid the onset of drought or nutrient stresses. Photosynthetic parameters were evaluated with 8-week-old fully expanded leaves.

### Chlorophyll Fluorescence and P700 Measurements

Both PSI and PSII parameters were monitored at 25°C by simultaneously recording chlorophyll fluorescence and P700 redox state with a Dual PAM-100 measuring system (Heinz Walz, Effeltrich, Germany). After light-adaptation at 611 μmol photons m^-2^ s^-1^ for 15 min to fully activate photosynthesis, light-adapted photosynthetic parameters were evaluated after exposure to each light intensity (923, 611, 421, 272, 172, 132, 94, and 59 μmol photons m^-2^ s^-1^) for 2 min.

The chlorophyll fluorescence parameters were calculated as follows: *F*_v_/*F*_m_ = (*F*_m_ -*F*_o_)/*F*_m_; *Y*(II) = (*F*_m_’ -*F*_s_)/*F*_m_’ (Genty et al., 1989); NPQ = (*F*_m_ - *F*_m_’)/*F*_m_’; qL = (*F*_m_’ -*F*_s_)/(*F*_m_’ -*F*_o_’) ×*F*_o_’/*F*_s_; *F*_o_’ = *F*_o_/(*F*_v_*/F*_m_ + *F*_o_/*F*_m_’) (Oxborough and Baker, 1997). *F*_o_ and *F*_m_ are the minimum and maximum fluorescence after dark-adaptation, respectively; *F*_o_’ and *F*_m_’ represent the minimum and maximum fluorescence after light adaptation, respectively; and *F*_s_ is the light-adapted steady-state fluorescence. Both *F*_o_ and *F*_m_ were determined after the plants were dark-adapted for 30 min before and after the high-light treatment was applied.

The PSI photosynthetic parameters were measured with a Dual PAM-100, based on the P700 signal (i.e., the difference in intensities of 830 and 875 nm pulse-modulated measuring light reaching the photodetector) (Klughammer and Schreiber, 2008). The P700^+^ signal (*P*) may vary between a minimum (P700 fully reduced) and maximum (P700 fully oxidized) level. We determined the maximum level (*P*_m_) by applying a saturating pulse (600 ms, 10,000 μmol photons m^-2^ s^-1^) after pre-illumination with far-red light, and then used *P*_m_ to estimate PSI activity (Huang et al., 2010; Brestic et al., 2015; Yamori et al., 2016; Takagi et al., 2017). The *P*_m_’ was determined similar to *P*_m_ but with actinic light instead of far-red light. The following calculations were made: quantum yield of PSI, *Y*(I) = (*P*_m_’ -*P*)/*P*_m_; the P700 oxidation ratio for a given actinic light, *Y*(ND) = *P*/*P*_m_; and the fraction of P700 that could not be oxidized by a saturating pulse in proportion to the overall P700, *Y*(NA) = (*P*_m_ - *P*_m_’)/*P*_m_.

Photosynthetic electron flows through PSI and PSII were calculated as: ETRII = *Y*(II) × PPFD × 0.84 × 0.5 and ETRI = *Y*(I) × PPFD × 0.85 × 0.5, where 0.5 was assumed to be the proportion of absorbed light reaching PSI or PSII, and 0.85 was assumed to be the absorbance (i.e., the fraction of incident light absorbed by the leaves). The value for CEF was estimated as ETRI – ETRII (Huang et al., 2012).

### Electrochromic Shift (ECS) Analysis

The ECS signal was monitored as the change in absorbance at 515 nm, which was measured with a Dual-PAM-100 that was equipped with a P515/535 emitter-detector module (Heinz Walz). After illumination at 54 μmol photons m^-2^ s^-1^ for 20 min, ECS decay was measured by switching off the actinic light for 30 s. The analysis of ECS dark interval relaxation kinetics was performed according to the methods of Sacksteder et al. (2001) and Takizawa et al. (2008). Total *pmf* was estimated from the total amplitude of the rapid decay of the ECS signal during a 300-ms dark interval. Slow relaxation of the ECS signal was used to recognize the proton gradient across the thylakoid membrane (ΔpH). The time constant for first-order ECS relaxation (τ_ECS_) is inversely proportional to the proton conductivity (*g*_H_^+^) of the thylakoid membrane through ATP synthase (Sacksteder and Kramer, 2000; Cruz et al., 2005). This allowed us to estimate *g*_H_^+^ as the inverse of the decay time constant [1/τ_ECS_]. The relative light-driven proton flux (*v*_H+_) was obtained by using the initial slope of the ECS relaxation.

### Photoinhibitory Treatments

After dark-adaptation for 30 min, *F*_v_/*F*_m_ and *P*_m_ were measured for intact leaves, which were then light-adapted at 611 μmol photons m^-2^ s^-1^ for 15 min. Afterward, light response curves were measured as described above before the actinic light was changed to 2258 μmol photons m^-2^ s^-1^. After exposure to this more intense illumination for 30 or 60 min, the light response curves were measured immediately. Finally, *F*_v_/*F*_m_ and *P*_m_ were evaluated after the leaves were dark-adapted for 30 min. To evaluate the ECS signal, we first exposed dark-adapted leaves to 59 μmol photons m^-2^ s^-1^ for 20 min to obtain the ECS parameters in unstressed leaves. Afterward, the same leaves were treated at 2258 μmol photons m^-2^ s^-1^ for 30 min. The ECS parameters were determined after adaptation at 59 μmol photons m^-2^ s^-1^ for 20 min. The level of actinic light was then changed to 2258 μmol photons m^-2^ s^-1^ or a period of 30 min before ECS parameters were again measured after the leaves were exposed to 59 μmol photons m^-2^ s^-1^ for 20 min.

### Statistical Analysis

All of the results were presented as mean values from five independent experiments. Independent *T*-test were used at a significance level of α = 0.05 (before/after) to determine whether the light treatments had a significant effect on photosynthetic parameters.

## Results

### Responses of PSI and PSII Activities to High Light

**FIGURE 1:**
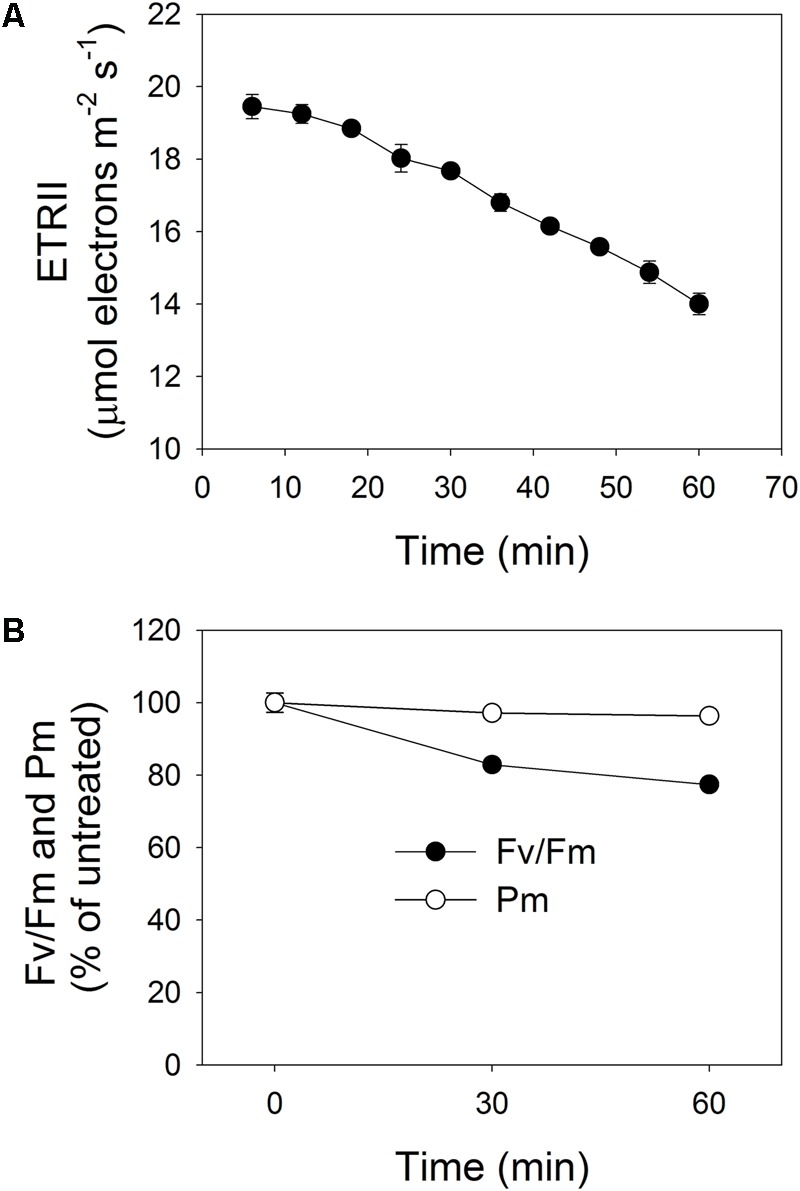
**(A)** Change in rate of electron flow through PSII (ETRII) during exposure to high light (2258 μmol photons m^-2^ s^-1^) for 60 min. After 25 min of light-adaptation at 611 μmol photons m^-2^ s^-1^ and measurement of light response curve (at Minute 16), actinic light intensity was changed to 2258 μmol photons m^-2^ s^-1^ and PSII parameters were recorded during next 60 min. **(B)** Changes in PSI and PSII activities after exposure to 2258 μmol photons m^-2^ s^-1^ for 30 and 60 min. Maximum quantum yield of PSII (*F*_v_/*F*_m_) represents PSII activity; maximum photo-oxidizable P700 (*P*_m_) represents PSI activity. Pre-treatment value for *F*_v_/*F*_m_ was 0.804. Values are means ± SE (*n* = 5).

To induce PSII photoinhibition, we exposed intact leaves of *P. notoginseng* to intense illumination, i.e., 2258 μmol photons m^-2^ s^-1^. Under such treatment, the value for ETRII gradually decreased, measuring 19.5 μmol electrons m^-2^ s^-1^ after 6 min and further declining to 14 μmol electrons m^-2^ s^-1^ after 60 min (**Figure 1A**). During that period, *F*_v_/*F*_m_ decreased by 17% after 30 min and by 23% after 60 min (**Figure 1B**), while *P*_m_ was reduced by only 3 and 4%, respectively (**Figure 1B**). These results demonstrated that this shade-adapted species *P. notoginseng* shows selective PSII photoinhibition under high-light stress.

### Effects of High-Light Treatments on Photosynthetic Electron Flow

**FIGURE 2:**
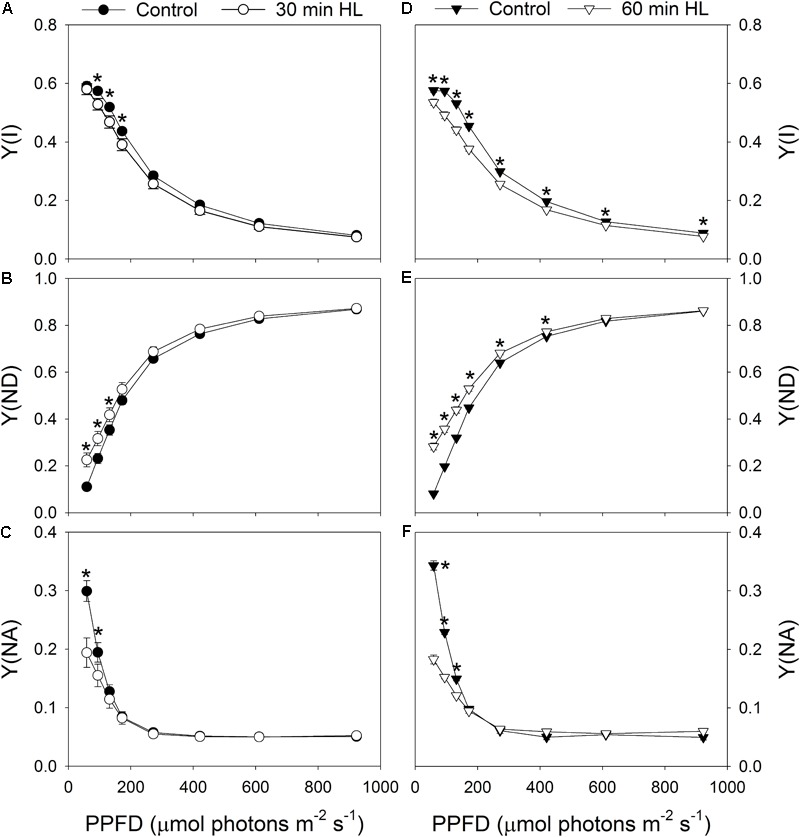
Effects of high-light treatments on light response changes in *Y*(I) **(A,D)**, *Y*(ND) **(B,E)**, and *Y*(NA) **(C,F)**. *Y*(I), quantum yield of PSI photochemical quenching; *Y*(ND), quantum yield of PSI non-photochemical energy dissipation due to donor-side limitation; *Y*(NA), quantum yield of PSI non-photochemical energy due to acceptor-side limitation. Values are means ± SE (*n* = 5). Asterisks indicate significant differences after high-light treatments.

Light response curves indicated that the value for *Y*(I) decreased slightly after 30 min of high-light treatment (**Figure 2A**). Meanwhile, the values for *Y*(ND) under light intensities below 132 μmol photons m^-2^ s^-1^ increased significantly (**Figure 2B**), and the values for *Y*(NA) under 59 μmol photons m^-2^ s^-1^ largely decreased (**Figure 2C**). After high-light treatment for 60 min, *Y*(I) decreased significantly under all light intensities (**Figure 2D**), *Y*(ND) increased significantly under light intensities below 421 μmol photons m^-2^ s^-1^ (**Figure 2E**), and *Y*(NA) decreased significantly under light intensities below 132 μmol photons m^-2^ s^-1^ (**Figure 2F**). This showed that the high-light treatment significantly affected the PSI redox state under low light.

**FIGURE 3:**
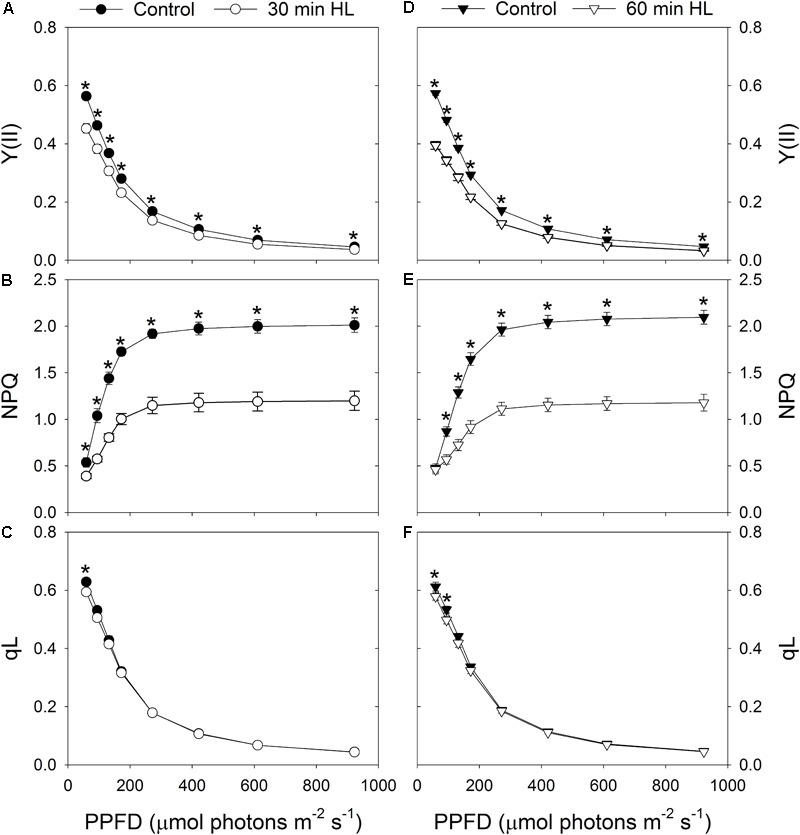
Effects of high-light treatments on light response changes in *Y*(II) **(A,D)**, NPQ **(B,E)**, and qL **(C,F)**. *Y*(II), effective quantum yield of PSII photochemistry; NPQ, non-photochemical quenching in PSII; qL, fraction of open PSII reaction centers. Calculations of NPQ and qL were based on values of *F*_o_ and *F*_m_ measured before and after treatment. Values are means ± SE (*n* = 5). Asterisks indicate significant differences after high-light treatments.

After high-light treatment for 30 min or 60 min, *Y*(II) decreased significantly under all light intensities (**Figures 3A,D**), and the capacity of NPQ largely declined (**Figures 3B,E**). We found it interesting that the redox state of *Q*_A_ (qL) was only slightly altered by these treatments (**Figures 3C,F**). This suggested that the ability of the leaves to utilize the product of LEF was hardly affected by exposure to intense illumination.

**FIGURE 4:**
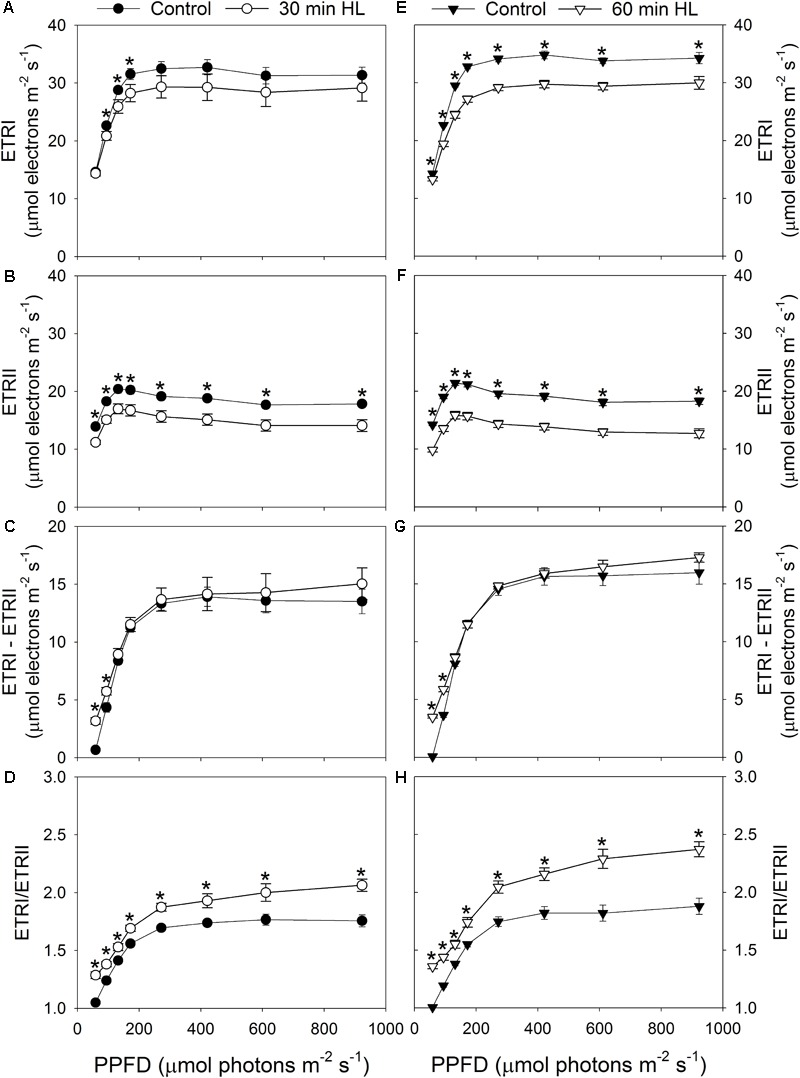
Effects of high-light treatments on light response changes in ETRI **(A,E)**, ETRII **(B,F)**, ETRI - ETRII **(C,G)**, and ETRI/ETRII ratio **(D,H)**. ETRI, the rate of photosynthetic electron flow through PSI. Values are means ± SE (*n* = 5). Asterisks indicate significant differences after high-light treatments.

After high-light treatment for 30 min, the values of ETRI under all light intensities decreased slightly (**Figure 4A**), while ETRII values under all light conditions decreased significantly (**Figure 4B**). By comparison, after 60 min of high-light treatment, both ETRI and ETRII decreased significantly regardless of intensity (**Figures 4E,F**). Although the capacity of LEF was depressed by these treatments (**Figures 4B,F**), the maximum value for CEF was only slightly changed (**Figures 4C,G**). Furthermore, CEF was stimulated significantly under low light intensities, i.e., 59 and 94 μmol photons m^-2^ s^-1^ (**Figures 4C,G**). Prior to high-light treatment, CEF was hardly activated at 59 μmol photons m^-2^ s^-1^. However, after high-light treatment for 30 to 60 min, CEF was significantly stimulated at that low level of light. Therefore, due to the decrease in ETRII, the ETRI/ETRII ratio increased significantly after high-light treatments, regardless of light intensity (**Figures 4D,H**).

### Effects of High-Light Treatments on Proton Motive Force

**FIGURE 5:**
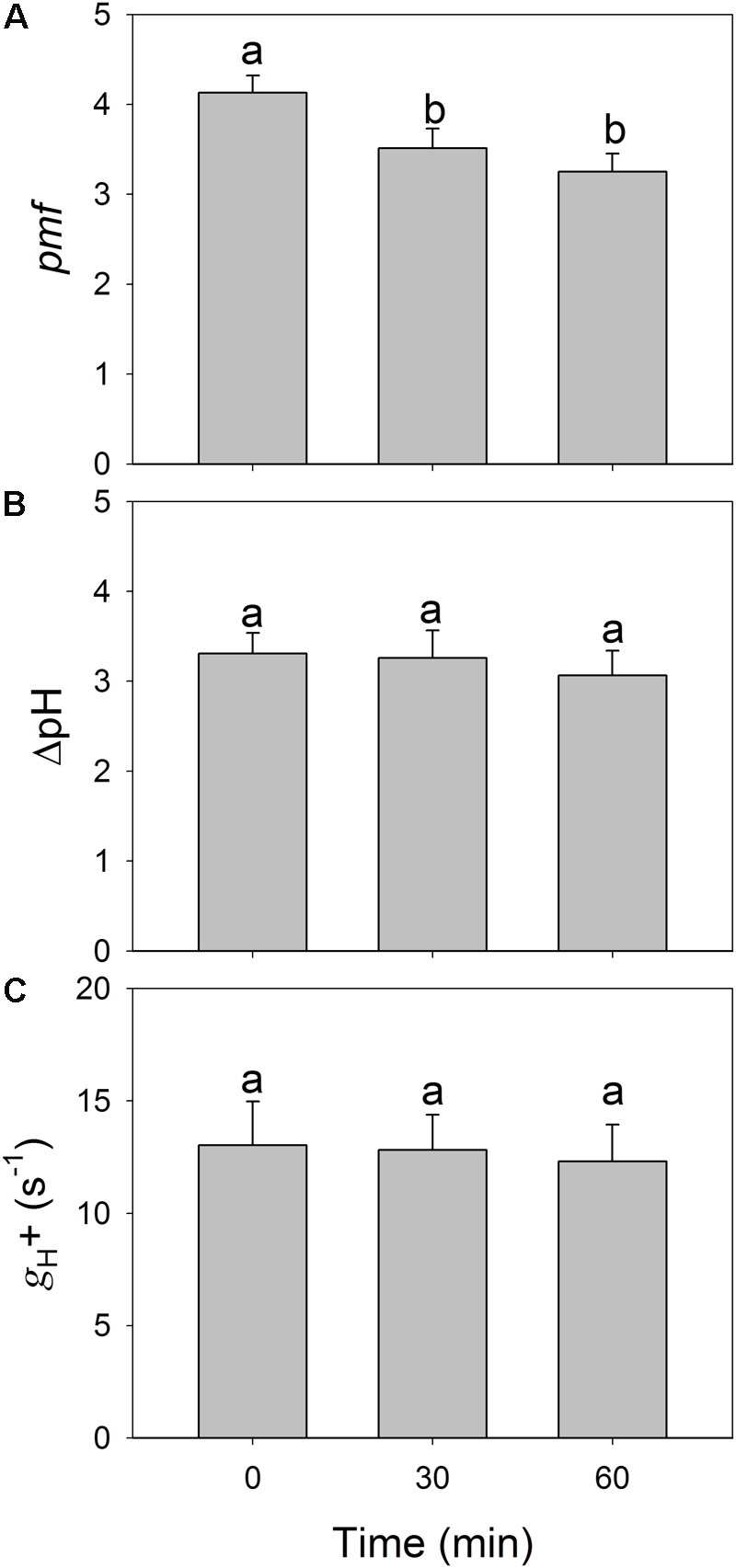
Changes in total proton motive force (*pmf*) **(A)**, proton gradient across the thylakoid membrane (ΔpH) **(B)**, and the proton conductivity of the thylakoid membrane (*g*_H_^+^) **(C)** after exposure to 2258 μmol photons m^-2^ s^-1^ for 30 and 60 min. All parameters were measured after adaptation at 54 μmol photons m^-2^ s^-1^ for 20 min. Values are means ± SE (*n* = 5). Different letters indicate a significant change after high-light treatments.

To examine whether the depression of LEF induced by high-light treatments is caused by enhanced lumenal acidification, we determined the ECS signal at 59 μmol photons m^-2^ s^-1^ before and after the treatments were applied. We found it interesting that total *pmf* decreased significantly after high-light treatments (**Figure 5A**) while ΔpH was not significantly changed (**Figure 5B**). The proton conductivity (*g*_H_^+^) of the thylakoid membrane was slightly altered before and after (**Figure 5C**), which suggested that the high-light treatments hardly affected the activity of chloroplastic ATP synthase under low light. These results indicated that the decline in LEF under low light could not be explained by the change in lumenal acidification.

**FIGURE 6:**
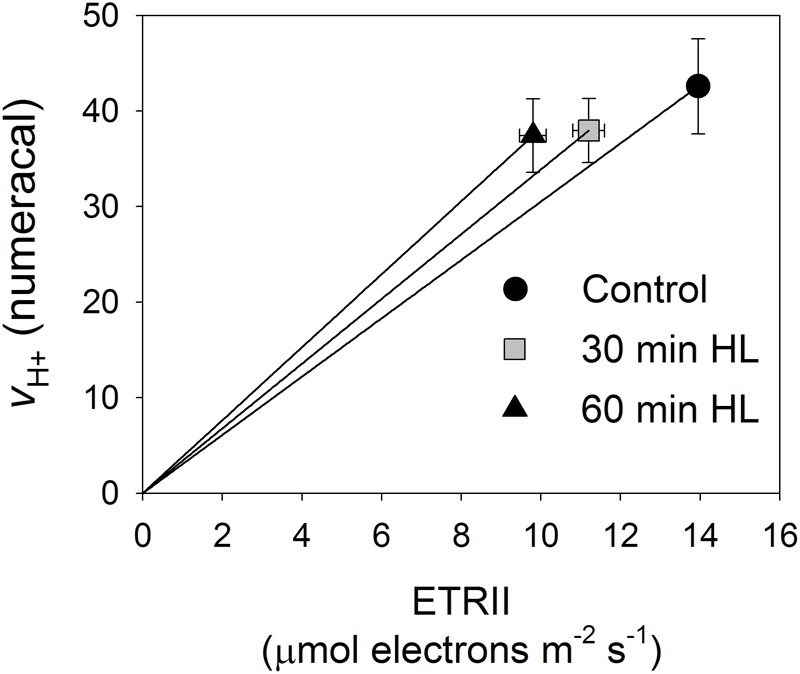
Relationship between relative light-driven proton flux (*v*_H+_) and ETRII. All measurements were made at 54 μmol photons m^-2^ s^-1^. Values are means ± SE (*n* = 5).

**Figure 6** shows the relationship between relative light-driven proton flux (*v*_H+_) and ETRII. This plot was developed to test for contributions from CEF because *v*_H+_ should reflect the proton flux generated by the operation of both CEF and LEF while the chlorophyll fluorescence-derived LEF parameter only measures electron transfer from PSII. After high-light treatments, leaves showed higher *v*_H+_ as a function of LEF, suggesting that CEF was stimulated under low light upon PSII photoinhibition.

### Effects of PSII Photoinhibition on LEF and P700 Oxidation Ratio at Low Light

**FIGURE 7:**
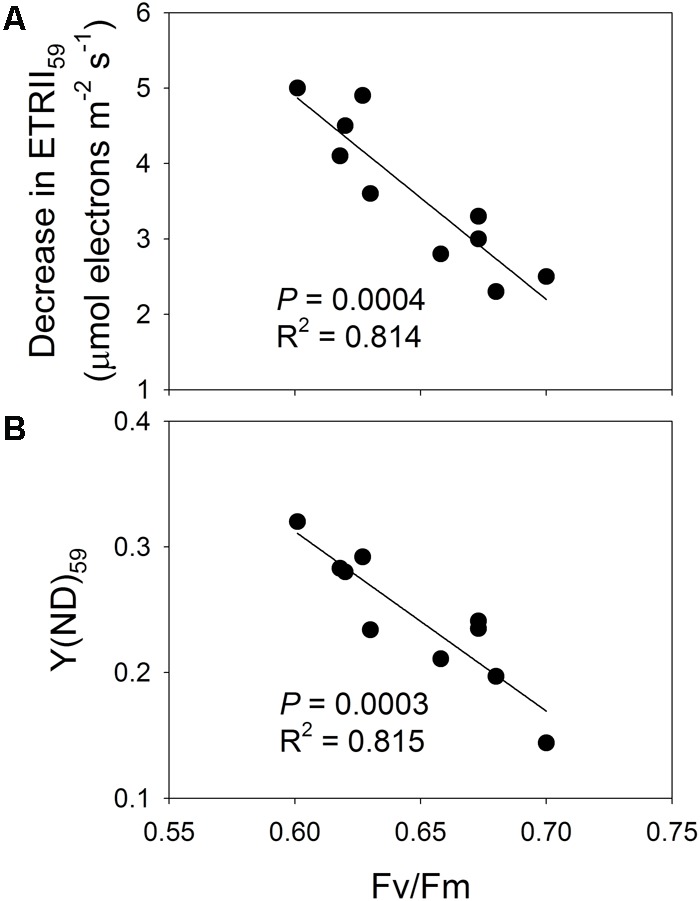
**(A)** Relationship between *F*_v_/*F*_m_ and decrease in ETRII_59_, and **(B)** relationship between *F*_v_/*F*_m_ and *Y*(ND)_59_. Decrease in ETRII_59_ represents reduction in ETRII at 59 μmol photons m^-2^ s^-1^ after high-light treatments; *Y*(ND)_59_ is value for *Y*(ND) measured at 59 μmol photons m^-2^ s^-1^ after treatments.

The effect of PSII photoinhibition on LEF under low light was further examined by pooling the data for *F*_v_/*F*_m_ and the decrease in ETRII at 59 μmol photons m^-2^ s^-1^ (ETRII_59_) after high-light treatments. Here, the value for *F*_v_/*F*_m_ was negatively correlated with the decrease in ETRII_59_ (**Figure 7A**), suggesting that residual PSII activity significantly affected the operation of LEF. That is, the number of PSII complexes was not sufficient to sustain maximum LEF under low light. We also examined the relationship between PSII photoinhibition and *Y*(ND) values at 59 μmol photons m^-2^ s^-1^ [*Y*(ND)_59_]. Our results indicated that *F*_v_/*F*_m_ was negatively correlated with *Y*(ND)_59_ (**Figure 7B**). Therefore, the extent of PSII photoinhibition significantly increased the P700 oxidation ratio under low light (**Figure 7B**).

## Discussion

### Different Responses of PSI and PSII to High-Light Treatments

Under strong illumination, excess absorbed light energy can cause PSII photoinhibition (Powles, 1984; Barber and Andersson, 1992; Aro et al., 1993a,b; Takahashi et al., 2009). High-light treatment induces a large decrease in PSII activity in the shade leaves of pioneer and late-succession tree species as well as other plants (Aro et al., 1993b; Kitao et al., 2000; Barth et al., 2001; Krause et al., 2004), while PSI activity is usually unaffected (Barth et al., 2001). However, PSI activity can also be sensitive to high-light stress in some shade-adapted tree species such as *Psychotria rubra, Psychotria henryi*, and *Nephrolepis falciformis* (Huang et al., 2015, 2016c,d, 2017, 2018). By comparison, we found that PSI activity was not susceptible to such stress in *P. notoginseng*, which indicated that this response is species-dependent and cannot be generalized. Because the response of photosynthesis to environmental stresses may be associated with the origin of a plant (Brestic et al., 2012), future studies should focus on a wide range of evolutionarily divergent species if we are to obtain a more comprehensive understanding of how PSI activity responds to high-light stress in shade-demanding species.

Photoinhibition occurs only under conditions in which the rate of photodamage exceeds the rate of its repair (Allakhverdiev et al., 2003, 2005, 2007, 2008; Allakhverdiev and Murata, 2004). After its reaction center is photodamaged, PSII can be rapidly and effectively repaired by replacing the damaged PSII proteins with newly synthesized proteins, primarily D1 protein (Allakhverdiev and Murata, 2004; Takahashi et al., 2009; Murata et al., 2012). The photorespiratory pathway and the Calvin-Benson cycle are important for protecting PSII against photoinhibition (Brestic et al., 1995; Lin et al., 2000; Murata et al., 2007; Takahashi et al., 2007). For the shade-adapted *P. notoginseng*, the relatively low capacity of ETRII indicates that this species has less capacity for the photorespiratory pathway and the Calvin-Benson cycle. As a result, under high-light stress, excess absorbed light energy induces the production of a large amount of reactive oxygen species that inhibit the fast recovery of its photodamaged PSII complexes (Nishiyama et al., 2001, 2004, 2006).

### PSII Photoinhibition Affects LEF Under All Light Intensities

Our results confirmed that LEF gradually decreased during exposure to a light intensity of 2258 μmol photons m^-2^ s^-1^. Furthermore, after exposure to this high level for 30 or 60 min, the rates of ETRII under all tested intensities decreased significantly. These results suggested that moderate PSII photoinhibition is accompanied by the depression of LEF under all light intensities in *P. notoginseng*, findings that are similar to those reported by Tikkanen et al. (2014). Although a decline in LEF upon PSII photoinhibition has been described by other researchers (Hikosaka et al., 2004; Hirotsu et al., 2005; Kornyeyev et al., 2006; Tikkanen et al., 2014), it is unclear whether that phenomenon is caused by a decrease in PSII activity or because lumenal acidification is enhanced. Electron transfer from PSII to PSI can be controlled by the Cyt *b_6_*/*f* complex, based on ΔpH (Nishio and Whitmarsh, 1993; Hope et al., 1994; Suorsa et al., 2012, 2016; Shikanai, 2014, 2016; Tikkanen and Aro, 2014). Over-acidification of the thylakoid lumen down-regulates LEF, thereby restricting overall plant growth (Livingston et al., 2010). Here, we found that ΔpH did not increase after the high-light treatments. Therefore, we cannot conclude that the reduction in LEF is associated with a change in the extent of lumenal acidification. Furthermore, the activity of thylakoid ATP synthase and qL were only slightly altered after high-light treatments. These findings strongly demonstrated that this depression of LEF was mainly caused by a decrease in PSII activity.

In studies that utilized the detached leaves of *A. thaliana*, results have been inconsistent about the influence of moderate PSII photoinhibition on LEF. For example, Kornyeyev et al. (2006) have shown that photoinhibition of less than 50% leads to a decrease in LEF at light intensities below 150 μmol photons m^-2^ s^-1^ but is not associated with LEF reductions at intensities above 150 μmol photons m^-2^ s^-1^. In contrast, Tikkanen et al. (2014) have reported that electron flow from PSII under high light is markedly diminished, i.e., by approximately 40%. Those studies also examined PSI photoinhibition due to either chilling treatment (Kornyeyev et al., 2006) or disruption of the PGR5-CEF pathway (Tikkanen et al., 2014). Because PSI activity plays important roles in photosynthetic regulation and energy balance (Munekage et al., 2002, 2004; Joliot and Johnson, 2011; Kramer and Evans, 2011; Walker et al., 2014, 2016), its photoinhibition has a serious impact on the rate of CO_2_ assimilation and, thus, LEF (Zivcak et al., 2015; Yamori et al., 2016). We also note that both of those studies involved detached leaves, which would affect stomatal conductance. As a result, PSI photoinhibition and the use of detached leaves would have presented important interactive factors that influenced LEF. In the present study, we excluded the effect of PSI photoinhibition by testing attached leaves, which also allowed us to avoid seeing a decrease in stomatal conductance during our treatments.

In detached leaves of *Capsicum annuum*, the light-saturated rates of oxygen evolution do not decrease when PSII activity declines by 40% (Lee et al., 1999). Accordingly, Lee et al. (1999) have assumed that PSII complexes occur in excess of the number needed to sustain light-saturated LEF by about 40%. We found here that PSII activity decreased by only 17%, which led to a significant reduction in LEF under all light intensities. Moreover, the experiments described by Lee et al. (1999) involved leaf disks for the photoinhibitory treatments and photosynthetic measurements, and the light-saturated rate of oxygen evolution could have been restricted by the *in vitro* state. Therefore, to overcome the influence of the *in vitro* state on stomatal conductance and LEF, we used attached leaves for all measurements. We were interested to learn that the extent of PSII photoinhibition was significantly correlated with the decrease in ETRII at 59 μmol photons m^-2^ s^-1^ after the high-light treatments. This indicated that the rate of LEF under low light is strongly determined by residual PSII activity. Therefore, PSII complexes may not occur in excess of the number needed to sustain maximum LEF in the shade-adapted *P. notoginseng*.

Shade-demanding plants usually grow slowly or die under high light (Krause et al., 2012). However, the underlying photosynthetic mechanisms are not clear. Species such as *P. notoginseng* are sensitive to high-light stress (Chen et al., 2016) and we found here that moderate PSII photoinhibition significantly depressed LEF under all light levels. Accordingly, after exposure to prolonged high-light stress, the rate of CO_2_ assimilation would be expected to show a large decrease due to severe PSII photoinhibition. When the carbon gain via photosynthetic CO_2_ assimilation cannot satisfy carbon consumption for respiration, plant growth ceases. Therefore, our present results provide new insight into the effect of high light on the growth of shade-demanding plants.

### PSII Photoinhibition Modifies PSI Redox State Under Low Light

The value calculated for *Y*(ND) under a low intensity of 59 μmol photons m^-2^ s^-1^ increased significantly after high-light treatments, and *Y*(ND) was negatively correlated with *F*_v_/*F*_m_. That is, PSII photoinhibition led to a significant increase in the P700 oxidation ratio. The value for *Y*(ND) depends upon two factors: (1) electron flow from PSII to PSI (Tikkanen et al., 2014; Huang et al., 2016b), and (2) the buildup of proton motive force (Munekage et al., 2002, 2004; Kanazawa et al., 2017; Takagi et al., 2017). When PSII activity is severely photo-damaged (i.e., PSII functioning is reduced to 40% of the control value), then *Y*(ND) under both low- and high-light intensities largely increases in *pgr5* plants of *A. thaliana* (Tikkanen et al., 2014). In *A. thaliana* mutants that have higher ATP synthase activity, the greater proton efflux from thylakoid lumen to stroma leads to a loss of lumenal acidification, causing a decline in *Y*(ND) and, thus, PSI photoinhibition (Kanazawa et al., 2017; Takagi et al., 2017). Although our study showed that *Y*(ND) increased at 59 μmol photons m^-2^ s^-1^, that response was not accompanied by a rise in ΔpH. We propose, therefore, that the increase in *Y*(ND) was mainly caused by depressed electron flow from PSII.

## Conclusion

We examined the effect of selective PSII photoinhibition on LEF in attached leaves of a shade-demanding species, *P. notoginseng*. Moderate PSII photoinhibition significantly depressed LEF under all light intensities. However, because PSI photoinhibition did not occur and lumenal acidification did not increase after high-light treatments, we suggest that this decrease in LEF was mainly caused by a significant decline in PSII activity. Therefore, this species might not have an adequate number of PSII complexes to sustain maximum LEF. These findings provide a new perspective on how intense illumination influences the growth of shade-adapted plants.

## Author Contributions

WH, S-BZ, and TL conceived and designed research and analyzed the data. WH conducted experiments and wrote the first draft of the manuscript, which was intensively edited by S-BZ and TL.

## Conflict of Interest Statement

The authors declare that the research was conducted in the absence of any commercial or financial relationships that could be construed as a potential conflict of interest.
